# Case of Mania

**Published:** 1800-12

**Authors:** 


### *Case of Mania.*

John Lauton, aged about 26 years, on the 11th of April, 1800, became deranged. For a fortnight before this period he had been much dejected, and, being under the influence of religious fear, had devoted the greateft part of his time to ferious reading. He had previoufly been in the habit of frequent intoxication, and his mother had been deranged at intervals for the laft twenty years. His pulfe and other functions, when I firft faw him, were natural; but he had been at times, for a few hours, very ungovernable. Religion was the topic on which he raved. I ordered a ftrait waiftcoat to be made ufe of, and prefcribed a folution of Antim. Tartarizat. which operated pretty well, when he had taken 8 grains. After the operation of the emetic, I directed him to take a few grains of camphor every four hours. On the 13th and 14th there appeared very little alteration in the fymptoms. His pulfe was about 80 in a minute, and of natural ftrength. Being coftive he had each day an enema adminiftered, but refufed to take any more medicines.

I defigned to put him under the influence of digitalis, but his attendants were unable to compel him to take it. I was furprifed to hear, on the 15th, of his death, which took place on the preceding evening. As I faw no fymptoms indicating danger during my laft vifit, I requefted leave to examine the body. On the 14th, in the morning, having become lefs untractable, his attendants had removed the ftrait waiftcoat. When I vifited him, about ten o'clock in the morning, I requefted his attendants again to apply the ftrait waiftcoat immediately; but this direction not being complied with, in the courfe of an hour he became fo unruly that fix men were unable to put on the ftrait waiftcoat; on this account they bound him in bed with cords. The efforts which he made to difentangle tangle himfelf, occafioned thofe parts of the body that came in contact with the cords, particularly about the thorax and thighs, to be much difcoloured.

### Appearances on Dissection.

On opening the head I found that the invefting membranes of the brain had no difeafed appearance, nor, indeed, did I obferve any unnatural appearance in the fubftance of the brain itfelf, until I divided a fmall portion lying immediately over the fella turcica; when I difcovered a quantity of florid blood mixed with air, in the form of froth, proceeding from a ruptured artery, a very fmall branch originating from the arterial circle of Willis. This extravafation, which did not exceed two or three drachms, feems to have been the immediate caufe of this man's death. Perhaps in this cafe, the preffure of the air which had efcaped from the artery, had been more injurious than the blood. The extravafation of two or three drachms of blood feems to be but a trifling caufe to produce fo great an effect. The body was opened about twenty-four hours after the patient's death. The fubftance of the brain, in this cafe, was not harder than ufual, that appearance being probably the effect of long-continued difeafe. On examining the thorax, a rib was found fractured by the cords; but the contents of the thorax were not difeafed. The heart feemed to be fomewhat fmaller than ufual.

Thofe alone who have practifed medicine will be able to form a juft idea of the difficulties which phyficians have to encounter, in combating the prejudices and affections of the fick and their attendants. The antipathy which the lower clafs of people have to the ufe of the ftrait waiftcoat is fuch, that often no reafoning, no entreaty, can prevail upon them to employ it, until, perhaps, the life of the patient, or his attendants, has been endangered; and even then, as foon as ever a lucid interval appears, the waiftcoat is thrown off, being confidered as a badge of infufferable difgrace. This is the fecond inftance of mania occurring in my practice within the laft eight months, which terminated fatally, apparently by binding the patient with cords. On this account, it is much preferable to fend maniacal patients to houfes of reception for this unfortunate clafs of people, than to leave them to the management of relatives and friends.

Far the greater proportion of cafes of mental derangement, which have fallen under my notice, have been excited by religious fear. The glowing and paffionate language, the rude and frantic geftures, the boifterous and thundering tone, in which eternal anathemas are dealt out from the pulpit; in which the terrors of hell are difplayed by ignorant enthufiafts amongft the fectaries, at once appal and paralyze the minds of their poor uninformed auditors. One idea only, the profpect of eternal punifhment, the image of hell before their eyes, occupies their judgment, rivets their attention. Confufion follows intenfity of thought, and derangement confufion. Several times has it been my lot to obferve the progrefs of caufe and effect above defcribed. In attending upon phthifical patients, I have frequently feen the few laft moments of thefe poor wretches embittered by the intrufion of fome religious fanatic, whofe gloomy picture of futurity has produced derangement.

